# Activity-Integrated Hidden Markov Model to Predict Calving Time

**DOI:** 10.3390/ani11020385

**Published:** 2021-02-03

**Authors:** Kosuke Sumi, Swe Zar Maw, Thi Thi Zin, Pyke Tin, Ikuo Kobayashi, Yoichiro Horii

**Affiliations:** 1 Interdisciplinary Graduate School of Agriculture and Engineering, University of Miyazaki, Miyazaki 889-2192, Japan; hc11093@student.miyazaki-u.ac.jp (K.S.); z3t1802@student.miyazaki-u.ac.jp (S.Z.M.); 2 Graduate School of Engineering, University of Miyazaki, Miyazaki 889-2192, Japan; pyketin11@gmail.com; 3 Field Science Center, Faculty of Agriculture, University of Miyazaki, Miyazaki 889-2192, Japan; ikuokob@cc.miyazaki-u.ac.jp; 4 Center for Animal Disease Control, University of Miyazaki, Miyazaki 889-2192, Japan; horii@cc.miyazaki-u.ac.jp

**Keywords:** calving, prediction, Hidden Markov Model, behavior changes

## Abstract

**Simple Summary:**

Dairy cows are known to become more active during the time calving approaches. Dairy farms provide individual calving pens to monitor the behavior of pregnant cows. Frequent posture changes such as alternating between lying and standing are good indicators that calving is imminent. In this paper, we aimed to determine how using these behavior changes or activities could help predict calving time. The activity monitoring video cameras in this study were located at a top corner of the calving pens so that the whole pens are visible. By processing the collected video sequences, the activities of pregnant cows three days before the calving were modeled in a Hidden Markov Model to predict the time when the calving event occurs. The experimental results show that the proposed method has promise.

**Abstract:**

Accurately predicting when calving will occur can provide great value in managing a dairy farm since it provides personnel with the ability to determine whether assistance is necessary. Not providing such assistance when necessary could prolong the calving process, negatively affecting the health of both mother cow and calf. Such prolongation could lead to multiple illnesses. Calving is one of the most critical situations for cows during the production cycle. A precise video-monitoring system for cows can provide early detection of difficulties or health problems, and facilitates timely and appropriate human intervention. In this paper, we propose an integrated approach for predicting when calving will occur by combining behavioral activities extracted from recorded video sequences with a Hidden Markov Model. Specifically, two sub-systems comprise our proposed system: (i) Behaviors extraction such as lying, standing, number of changing positions between lying down and standing up, and other significant activities, such as holding up the tail, and turning the head to the side; and, (ii) using an integrated Hidden Markov Model to predict when calving will occur. The experiments using our proposed system were conducted at a large dairy farm in Oita Prefecture in Japan. Experimental results show that the proposed method has promise in practical applications. In particular, we found that the high frequency of posture changes has played a central role in accurately predicting the time of calving.

## 1. Introduction

The calving process is one of the most stressful and painful experiences for a dairy cow to deal with during the production cycle. Dystocia [1] is a prolonged, abnormal, or difficult birth, which is a serious issue for dairy farmers because it can cause economic loss, and dealing with it is labor-intensive. Such difficulties result from prolonged spontaneous calving or prolonged or severe assisted extraction. Therefore, farm management personnel require accurate and reliable systems for predicting when calving will occur, helping decide when to provide assistance. Naturally, cows are vulnerable when calving, due to the changes and stress they experience. Predicting the exact time of calving, as well as specific calving behaviors, requires continuous monitoring. Precision cow monitoring technologies such as image processing techniques can provide visual information for the assessment of calving behaviors, and help minimize difficulties. According to some research findings [1,2,3], the rate of mortality due to difficult calving reaches 35% at the international level. Jensen described that managing the calving process is the most important area of dairy research, specifically for preventing long-term illness [4]. Most dairy farmers and observers know that cows become restless immediately before calving. According to some previous research works [5,6,7], this restlessness is characterized by irregular behavior such as frequent changes in posture, holding up the tail, turning the head backwards, lying, standing, and bouts of frequently changing position between lying down and standing up. In addition, changes in the behavioral patterns of dairy cows have been used by farmers and animal doctors to detect poor health, and determine phases in the estrus cycle. Among the many behavior patterns, those of lying down, and bouts of changing position between lying down and standing up, rank high as subjects of study [6,7,8]. However, even after years of research, these behaviors are not fully understood. Exactly how these behaviors relate to the calving process, and to other aspects of dairy farming, must be investigated.

In this paper, we integrate into a Hidden Markov Model (HMM) the activities of lying down, standing up, bouts of frequently changing position between lying down and standing up, as well as the proportion of time spent in changing posture, and then perform a modified Viterbi Algorithm to predict when calving will occur.

In order to predict when calving begins, different researchers have proposed various methods and technologies, including monitoring through the use of wearable sensors [9,10,11,12], observing changes in body temperature [13], and a variety of techniques using biosensors for monitoring tail elevations [14,15]. Mee and English discuss methods and devices developed for calving prediction, presenting a good summary of the current status of methodologies and technologies in this area of research [5].

Among various findings, the methods can be categorized into three groups. They are the prediction of calving time based on (i) hormonal changes, (ii) clinical signs, and (iii) behavioral changes just before calving. According to Mee and English, the systems based on behavioral changes seem to be the most promising, since significant changes can be monitored within a day of calving. However, predicting the exact starting time is still difficult.

**Figure 1:**
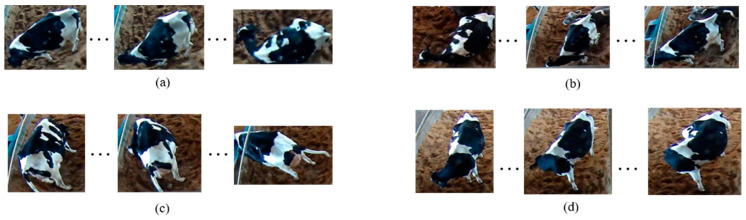
Bovine activities: (**a**) Lying down, (**b**) standing up, (**c**) holding up the tail, (**d**) turning the head backwards.

Recently, as discussed in [16], the authors described rumination and feeding behavior around calving and the time spent on these behaviors are monitored and analyzed. The authors in [8] predicted calving time using data from a RumiWatch noseband-sensor (ITIN + HOCH GmbH, Liestal, Switzerland) and a 3D-accelerometer. The authors stated that lying bouts increased and rumination decreased in primiparous and multiparous cows, respectively. Some studies have involved predicting calving time using activities such as trying to hide [17], moving the tail [18], walking aimlessly [19], turning the head towards the abdomen [20], reducing time spent in rumination [6], reducing time spent lying down, sniffing the ground [21], and frequently changing posture [22]. Figure 1 shows a variety of activities, such as standing up, lying down, holding up the tail, and turning the head backwards.

Now let us see what kinds of methods and techniques have been used for calving prediction in the environment of a maternity barn. Most prediction methods are developed through establishing a mathematical model. These methods are based on standard data analysis: Data preparation and logistic regression, artificial neural networks, and use of a support vector machine. Fenlon et al. created the four machine learning techniques: Multinomial regression, decision trees, random forests, and neural networks for predicting three levels of calving difficulty (unassisted, slight assistance, and considerable or veterinary assistance) [23]. Included in their analysis were variables related to parity, genetics, BCS, breed, previous calving experience, previous reproductive events, and the calf. Moreover, there are three machine learning techniques such as random forest, linear discriminant analysis, and neural network were used for calving time prediction and their method has developed a daily calving prediction model and bihourly calving models [7].

Most calving prediction methods developed so far require visual observations by experts in one way or another. All researchers attempted to develop automated systems that predict calving times, rather than require manual processes by farming personnel or veterinarians [24]. In our previous work [25], the analysis on cow’s calving process is performed by using the motion features such as tail up, increasing movement, repeated transitions and so on. Sumi et al. proposed automatic cow behavior detection by using image processing and computer vision techniques to implement in cow calving monitoring system [26]. The frequency of standing bouts and lying bouts were considered as the variables to predict calving. To extract cow regions from input images recorded in delivery barns, the authors in [27] proposed a background modelling method first using a Temporal Average filter and then the Gaussian Average method for updating the background model. In order to analyze the behaviors of a cow, the system must detect the cow regions. In our previous paper [28], patterns of cow motions are analyzed to predict calving time. These patterns included transition states such as standing-to-lying and lying-to-standing. In this paper, we mainly propose a promising new integrated HMM based on behavior changes and the duration of time spent lying down when calving is imminent. The proposed HMM is a new approach in calving time prediction. Although many other approaches such as machine learning techniques haven been appeared, our approach is much easier and not much data are needed for the training process. The rest of the paper is organized as follows: Section 2 describes the main contributions of the paper related to the proposed methodology, and Section 3 provides the experimental results. We conclude the paper in Section 4.

## 2. Proposed Dairy Cow Calving Time Prediction

**Figure 2:**
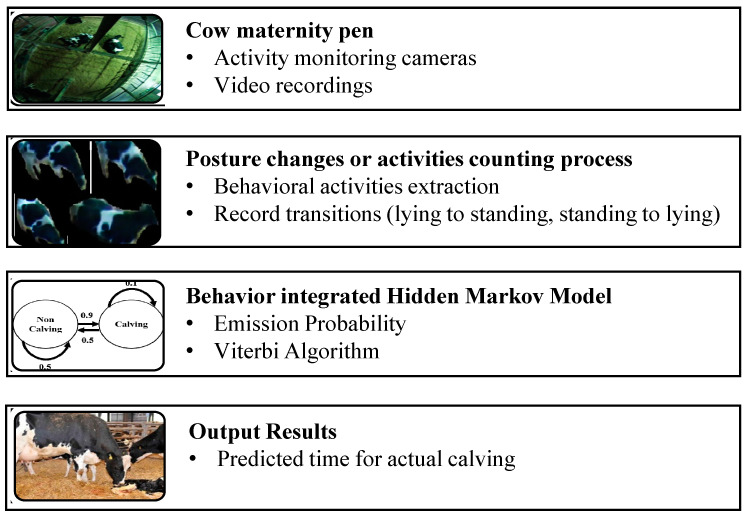
Overview of proposed dairy cow calving time prediction.

In this section, we propose an activity-integrated HMM to predict when calving will occur. Figure 2 provides an overview of the proposed model. The system is composed of two functional units: (1) Behavior classification and counting of each behavior, (2) the activity-integrated HMM Markov Model, as well as the final pipeline of the decision-making unit (results output).

Video recordings were taken of cow behavior during the days and nights before actually calving. Our system focuses on behavior such as time spent lying down, and changes in posture such as bouts of changing position between lying down and standing up. In addition, we also recorded tail movements, and head turns. Such types of data are collected by human observers. In our proposed system, we use the terms standing bouts, referring to the transition from lying down to standing up, and lying bouts, referring to the transition from standing up to lying down. The step by step processes for our calving time prediction model are described in the following sub-sections.

### 2.1. Activity Observation

Generally, data on this behavior are collected three to seven days ahead of the expected calving time, defined as the cow’s behavior, with each type of behavior serving as an independent variable. The behavior collected data for 3 days before calving is sufficient to make prediction while compared to behavior collected data for 7 days. Therefore, in our case, we only used data from 3 days prior to calving. Calving time is considered a dependent variable. In this paper, activities such as standing up, lying down and changes in posture are considered a prelude to calving. These behavior observations were performed on 25 cows by human observers.

### 2.2. Data Summarization

Video recordings for the behaviors of the individuals were made over a duration of three days before the expected calving dates. This paper, reports on the results of an experiment performed on 25 dairy heifers from the farm in Oita prefecture, Japan. The pregnant heifers were continuously video-monitored by a 360-degree GV-FER5700 camera (GeoVision Inc., Taiwan, China) located at the center of the barn providing the best possible view of the whole barn. The camera has a resolution of 2560 × 2048 pixels, recording at 30 frames per second.

The camera was placed 3 m above the floor in each loose housing calving pen. Cows were free to move about within a space measuring approximately 7 × 7 square meters, on a floor covered with sawdust for bedding. In our experiments, we collected data on 25 pregnant heifers, 19 Holstein first-calf heifers and 6 Brown-Swiss first-calf heifers, which calved from November to December in 2017. Parturitions were observed at ages between 21 and 26 months. These heifers became pregnant by embryo transfer. The recipient heifers were transferred in vivo-developed embryos obtained from Japanese black cows. The donor Japanese black cows were artificially inseminated with frozen–thawed semen from Japanese black bulls to collect embryos to transfer after superovulation treatment. No twin or triplet births occurred during this study. Group feeding system for two to four animals were used during this study depending on the timing of parturition of each heifer. They were fed with Total Mixed Ration (TMR) twice daily which was calculated depending on the prospected average milk yield (35 kg/day) after parturition and body weight estimated. Clean water and mineral supplements were available ad libitum during the entire period. Information on recording start time and calving data for the 25 cows are shown in Table 1.

The categories of behavior described in Table 2 are used to observe the activities of heifers over the last 72 h before delivery. Here we assume that the delivery time extends to the moment when the calf was fully expelled. These observations were recorded using the instantaneous sampling recording rule. The camera started video recording every 5 min, and the time spent on each was aggregated on an hourly basis as described in Table 3.

The data used in our experiment were collected by human observers through video monitoring of individual heifers in the calving pen. Standing bouts (changing from lying to standing) and of lying bouts (from standing to lying) were counted to determine the number of posture changes from the video sequences. We then obtain the count of posture changes by combining the number of standing bouts and lying bouts every hour. Activities related to standing include standing still, walking and eating, and those related to lying include lying while inactive, and ruminating while lying.

### 2.3. Integrated Hidden Markov Model for Calving Time Prediction

HMM is a class of statistical modeling for sequential data, in most instances related to systems evolving over time. The system of interest is modeled using a state process or system process which evolves dynamically such that future states depend on the current state. The posture changes before calving can naturally be described by such a process evolved over time. Thus, in the process of predicting calving time, we assume that the system includes two types of states, non-calving (NC) state and calving (C) state which can be difficult to observe directly but that can be uncovered by using some information about non-calving state.

Activity data collected as described above is integrated into a HMM to predict when calving will occur. This model includes two hidden states to discover: NC and C. Our objective is to discover the time T at which calving occurs. Although these two hidden states are not directly observable, we can observe activities associated with these states in the days preceding a calving event. The probability of an observed activity being related to a hidden state is known as an emission probability.

Without loss of generality, as a univariate activity (say changes in postures), the number of changes in postures can be divided into three levels namely Low (L), Medium (M), and High (H). To make the problem specific, the levels of posture changes will be defined as follows. Suppose the collected sequence of posture changes is *x_1_*, *x_2_*, *x_3_*, …, *x_N_*, where *N* stands for time (can be minute, hour or any duration). First calculate the sample mean (*μ*) and sample standard deviation (*σ*). Definitions of Activity Levels are as follows:

1. If the observed value for posture changes is less than the sample mean (*μ*) then the activity is at a low level, and denoted by *L*.
2. If the observed value for posture changes lies between (*μ*) and (*μ* +*σ*) then the activity is at a medium level, and denoted by *M*.
3. If the observed value for posture changes is greater than (*μ* +*σ*) then the activity is at a high level, and denoted by *H*.

**Figure 3:**
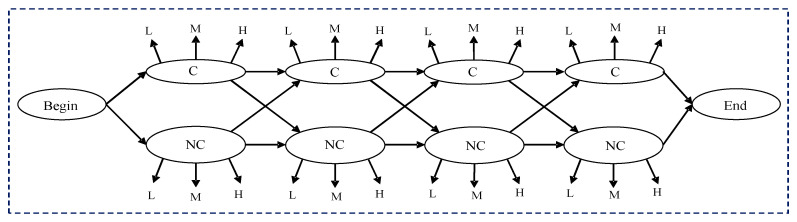
Illustration of Integrated Hidden Markov Calving Time Prediction Model.

For each of two states (NC, C), the defined three levels (L, M, H) are observable, and their corresponding probabilities will become the emission probabilities in the HMM. Thus, the proposed calving time prediction model can be described as shown in Figure 3.

Now we summarize the prediction model into usual notation of HMM.

(1) $$A=\left[\begin{array}{cc} a_{11} & a_{12}\\ a_{21} & a_{22} \end{array}\right]$$

where *a*_11_ = transition probability from non-calving state to non-calving state,
*a*_12_ = transition probability from non-calving state to calving state,
*a*_21_ = transition probability from calving state to non-calving state,
*a*_22_ = transition probability from calving state to calving state.

We assume that the initial probabilities for the states are given by:

(2) $$\Pi =\left[\;\Pi_{1}\;\Pi_{2}\right]\;,$$

$$\Pi_{1}+\Pi_{2}=1$$

where, Π_1_ = probability of non-calving,
Π_2_ = probability of calving

The Low, Median, and High activity levels (L, M, H) are observable states which we can learn from the video sequences. The corresponding probabilities of L, M, and H are known as emission probabilities. In a matrix form, we denote emission probabilities as B. In our case the emission probabilities will derive from the activity level data and denoted by B as follows:

(3) $$B=\left[\begin{array}{ccc} P\;(L|\mathrm{NC}) & P\;(M|\mathrm{NC}) & P\;(H|\mathrm{NC})\\ P\;(L|C) & P\;(M|C) & P\;(H|C) \end{array}\right].$$

In formal notation, the complete HMM becomes as follows:

(4) λ=(A, B, Π).

The most likely state was then calculated using transition matrix A and emission probability matrix B from the fitted logistic regression model using the Viterbi algorithm. The Viterbi algorithm is a recursive method to calculate the most likely path of hidden states given observed probabilities and state transition probabilities.

### 2.4. Methodology Implementation Procedure

The experimental videos were collected for 25 heifers. From these videos, posture changes were then counted, referring to changes from lying to standing, and from standing to lying. We organized the data on an hourly basis. The sample data is shown in Table 3. In our paper, we have utilized cow behavioral observations such as posture changes done by human observers for 72 h video before giving birth.

The sample mean and standard deviation of the frequency of posture changes are calculated from columns 2, 5, and 8 in Table 3. Table 4 shows the results for mean and standard deviation. To define the activity levels, the numbers for posture changes in the columns (2, 5, 8) are compared with the values of *µ* and *µ* + *σ* described in Table 3. Then, we obtain the activity levels for the columns (3, 6, 9) in Table 3:

The activity levels are defined as follows:

- L: *n* < *µ*,
- M: *µ* < *n* < *µ* + *σ*,
- H: *n* > *µ + σ*

where, *n* = posture changes, *µ* = 1.931 and *µ* + *σ* = 3.897.

**Figure 4:**
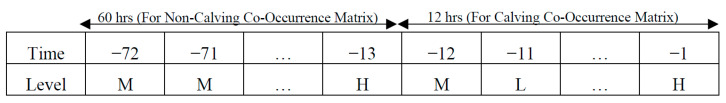
Sequence of Activity Level.

The co-occurrence matrix for NC and C states are calculated from activity levels (L, M and H) in data collected over 72 h data as seen in columns 3, 6, and 9 from Table 3. We then count the number of occurrences of the combinations (L, L), (L, M), (L, H), …, (H, H), totaling 9 possible combinations, and obtaining a 3 × 3 co-occurrence matrix for NC and C state. The sequence of labels for L, M and H from Table 3 can be seen as shown in Figure 4.

In order to derive the co-occurrence matrices for NC and C, we first assume that, the first 60 h to be a NC state and the final 12 h to be a C state.

For NC states, the co-occurrence matrix is given by:

$$\mathrm{NC}=\left[\begin{array}{ccc} 16 & 9 & 3\\ 10 & 10 & 4\\ 2 & 4 & 1 \end{array}\right].$$

And for C state, the co-occurrence matrix is:

$$C=\left[\begin{array}{ccc} 2 & 1 & 0\\ 1 & 3 & 2\\ 0 & 1 & 1 \end{array}\right].$$

By normalizing rows of each co-occurrence matrix NC and C, we obtain the corresponding probability matrices, respectively denoted by P and Q for non-calving and calving states.

$$P=\left[\begin{array}{ccc} 0.571 & 0.321 & 0.107\\ 0.416 & 0.416 & 0.167\\ 0.286 & 0.571 & 0.143 \end{array}\right]$$

$$Q=\left[\begin{array}{ccc} 0.667 & 0.333 & 0.000\\ 0.167 & 0.500 & 0.333\\ 0.000 & 0.500 & 0.500 \end{array}\right]$$

**Figure 5:**
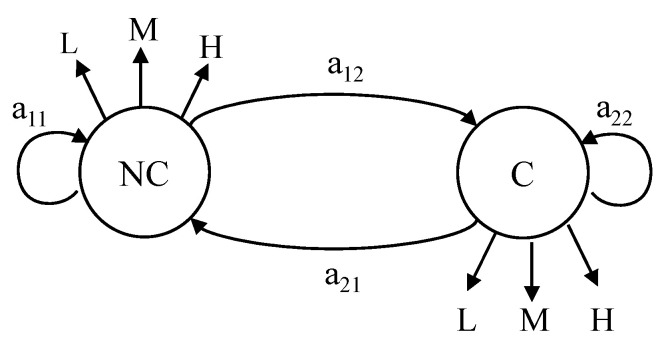
The state diagram for non-calving (NC) state and calving (C).

We then calculate the stationary distributions by solving the above equations of P and Q. We obtain the stationary distribution for P as (0.475, 0.393, 0.135), and the stationary distribution for Q as (0.231, 0.462, 0.308). These two distributions will be used as emission probabilities B for NC and C states, respectively. Also, from Table 3, we obtain the transition probability matrix A. The illustration of state diagram for NC and C is shown in Figure 5.

Initially we consider the first 60 h as NC state and C state will begin in next one hour. The total time taken for NC to NC and NC to C is 61 h. The transition probability from NC to NC as *a*_11_ (60/61 = 0.984) and NC to C as *a*_12_ (1/61= 0.016). In similar ways, the remaining 12 h is in C state and it will to NC state in next one hour. The transition probability from C to NC as *a*_21_ (1/13 = 0.077) and C to C as *a*_22_ (12/13 = 0.923). The transition probability matrix for the two Hidden States: NC and C states, shown as follows:

$$A=\left[\begin{array}{cc} 0.984 & 0.016\\ 0.077 & 0.923 \end{array}\right]$$

Now the HMM is completely defined with transition matrix A. The emission probability matrix B from stationary distributions is

$$B=\left[\begin{array}{ccc} 0.472 & 0.393 & 0.135\\ 0.231 & 0.462 & 0.308 \end{array}\right]$$

Here, the initial probability to be considered as Π= [0.5, 0.5].

## 3. Experimental Results

In order to obtain the experimental results, we apply the Viterbi algorithm to the HMM developed in Section 2. Consequently, we have the most likely state sequence for a given observed sequence as shown in Table 5.

The last row of Table 5 showed that the calving occurs during last three hours, which agrees with the time of the actual occurrence of calving. Among these 25 heifers, we randomly selected 15 heifers to estimate HMM parameters, and testing proceeded on the remaining 10 heifers. The performance analysis on testing data of 10 heifers is shown in Table 6. Overall, our system achieved the average sensitivity of 91.05% and average precision of 93.28% on testing data.

The actual time of calving lies one standard deviation away from the sample. Thus, the error term is absolute value of difference (3 − (*µ* + *σ*)) = 0.897. Therefore, the error percentage is (0.897)/3 which is equal to 0.299. This gives a mean square error of (0.299)^2^ = 0.089401. We can conclude that the overall accuracy rate is (1 − 0.089401) × 100 = 91.0599, a little over 91%.

## 4. Conclusions

We propose an activity-integrated HMM for predicting when calving will occur. For developing our method in this study, we only used data for posture changes such as lying bouts and standing bouts among many other features such as tail up and increasing movements. We expect that prediction accuracy will improve by using data for additional activities such as time spent lying down, rumination, tail movements, and head movements. To conduct the real time calving prediction system, real time cow identification, activity observation and prediction model will then be combined to become a complete system. Much research remains to be done in the future, by collecting and testing additional data.

## Figures and Tables

**Table 1:** Calving data information for 25 pregnant heifers.

ID	Recording Started Date and Time		Calving Date and Time	
	(mm/dd/yy)	(h/min/s)	(mm/dd/yy)	(h/min/s)
1	11.29.2017	(00:00:00)	12.02.2017	(12:32:50)
2	11.26.2017	(17:10:00)	11.29.2017	(17:10:00)
3	11.26.2017	(19:35:00)	11.29.2017	(19:35:00)
4	12.04.2017	(10:05:00)	12.07.2017	(10:06:35)
5	11.30.2017	(15:10:00)	12.03.2017	(15:10:00)
6	11.30.2017	(21:15:00)	12.03.2017	(21:15:00)
7	12.04.2017	(10:10:00)	12.07.2017	(10:13:00)
8	12.04.2017	(16:05:00)	12.07.2017	(16:09:40)
9	12.04.2017	(14:00:00)	12.06.2017	(20:10:00)
10	12.11.2017	(06:00:00)	12.14.2017	(05:58:50)
11	12.06.2017	(10:00:00)	12.08.2017	(03:25:00)
12	12.12.2017	(04:50:00)	12.15.2017	(04:53:09)
13	12.07.2017	(17:20:00)	12.10.2017	(17:20:00)
14	12.13.2017	(21:00:00)	12.16.2017	(21:03:29)
15	12.16.2017	(21:55:00)	12.19.2017	(21:55:00)
16	12.14.2017	(17:15:00)	12.17.2017	(17:19:20)
17	12.17.2017	(06:10:00)	12.20.2017	(06:10:00)
18	12.14.2017	(16:15:00)	12.17.2017	(16:17:12)
19	12.17.2017	(09:50:50)	12.20.2017	(09:50:00)
20	12.15.2017	(01:25:00)	12.18.2017	(01:25:21)
21	12.17.2017	(12:15:00)	12.20.2017	(12:15:00)
22	12.09.2017	(17:15:00)	12.12.2017	(17:15:00)
23	12.01.2017	(10:25:00)	12.04.2017	(10:25:18)
24	12.03.2017	(02:40:00)	12.06.2017	(02:41:22)
25	11.29.2017	(00:30:00)	12.02.2017	(00:30:00)

**Table 2:** Activity variable descriptions.

Variable Name	Unit Measure	Descriptions
Lying Time	Minute/Interval	The time in minutes that a cow has spent lying with in a predefined interval
Standing Time	Minute/Interval	The time in minutes that a cow has spent standing with in a predefined interval
Lying Bouts	Number/Interval	The number of transitions from standing to lying during the interval
Standing Bouts	Number/Interval	The number of transitions from lying to standing during the interval

**Table 3:** Sample posture changes data in experiment.

Cow ID 1								
Time	Posture Changes	Activity Level	Time	Posture Change	Activity Level	Time	Posture Change	Activity Level
−72	2	Mid	−48	2	Mid	−24	3	Mid
−71	2	Mid	−47	4	High	−23	3	Mid
−70	5	High	−46	2	Mid	−22	4	High
−69	6	High	−45	3	Mid	−21	2	Mid
−68	1	Low	−44	1	Low	−20	4	High
−67	2	Mid	−43	2	Mid	−19	2	Mid
−66	1	Low	−42	1	Low	−18	2	Mid
−65	0	Low	−41	0	Low	−17	0	Low
−64	0	Low	−40	1	Low	−16	0	Low
−63	2	Mid	−39	2	Mid	−15	0	Low
−62	2	Mid	−38	2	Mid	−14	0	Low
−61	2	Mid	−37	1	Low	−13	4	High
−60	2	Mid	−36	4	High	−12	2	Mid
−59	1	Low	−35	1	Low	−11	0	Low
−58	2	Mid	−34	2	Mid	−10	1	Low
−57	2	Mid	−33	1	Low	−9	1	Low
−56	1	Low	−32	0	Low	−8	2	Mid
−55	0	Low	−31	0	Low	−7	2	Mid
−54	0	Low	−30	0	Low	−6	2	Mid
−53	0	Low	−29	1	Low	−5	6	High
−52	1	Low	−28	4	High	−4	2	Mid
−51	1	Low	−27	2	Mid	−3	2	Mid
−50	2	Mid	−26	3	Mid	−2	5	High
−49	1	Low	−25	0	Low	−1	13	High

L: Low, M: Medium, H: Height.

**Table 4:** Sample mean and standard deviation.

Sample Mean *µ* of posture change frequency	1.931
Standard Deviation *σ* of posture change frequency	1.967
(*µ* + *σ*)	3.897
(*µ* + 2*σ*)	5.864

**Table 5:** The most likely state sequence.

Actual Time	3 h Interval 1–24	Activity	States	Actual Time	3 h Interval 1–24	Activity	States
−72	−24	L	NC	−36	−12	L	NC
−69	−23	L	NC	−33	−11	L	NC
−66	−22	L	NC	−30	−10	L	NC
−63	−21	L	NC	−27	−9	L	NC
−60	−20	L	NC	−24	−8	M	NC
−57	−19	L	NC	−21	−7	L	NC
−54	−18	L	NC	−18	−6	L	NC
−51	−17	L	NC	−15	−5	L	NC
−48	−16	L	NC	−12	−4	L	NC
−45	−15	L	NC	−9	−3	L	NC
−42	−14	L	NC	−6	−2	M	NC
−39	−13	L	NC	−3	−1	H	C

L: Low, M: Medium, H: Height, NC: Non-calving, C: Calving.

**Table 6:** Performance analysis for 10 heifers.

No.	Sensitivity (%)	Precision (%)
1	91.30	95.45
2	86.96	95.24
3	90.63	87.88
4	95.83	100
5	91.67	100
6	90.48	86.36
7	91.67	100
8	91.67	100
9	85	80.95
10	95.24	86.96
Average	91.05	93.28

## Data Availability

The data presented in this study are available on request from the corresponding author. The data are not publicly available due to patent pending.
